# Wireless nonresonant stimulation of neurons on a magnetoelectric film surface

**DOI:** 10.1126/sciadv.adx6829

**Published:** 2025-10-17

**Authors:** Asli Aydin, Ali Jahanshahi, Pouria Esmaeili-Dokht, Mertcan Han, Gaurav Gardi, Yan Yu, Ren Hao Soon, Yasin Temel, Metin Sitti

**Affiliations:** ^1^ Physical Intelligence Department, Max Planck Institute for Intelligent Systems, 70569 Stuttgart, Germany.; ^2^ Department of Neurosurgery, Maastricht University Medical Centre, Maastricht, Netherlands.; ^3^ Netherlands Institute for Neuroscience, Royal Netherlands Academy of Arts and Sciences, Amsterdam, Netherlands.; ^4^ Institute for Biomedical Engineering, ETH Zürich, 8092 Zürich, Switzerland.; ^5^ Faculty of Medicine, Istanbul Atlas University, Istanbul, Turkey.; ^6^ School of Medicine and College of Engineering, Koç University, 34450 Istanbul, Turkey.

## Abstract

Wireless neural interfaces are emerging as a minimally invasive treatment option for neurological disorders. Among the wireless technologies, magnetically powered systems are effective for targeting deep brain sites. However, dependence on high-frequency electromagnetic fields in such systems limits their safe implementation. In this study, we demonstrate the use of millimeter-scale magnetoelectric (ME) films as a direct neural interface for wireless neurostimulation, powered by static and alternating magnetic fields in the nonresonant regime (10 hertz). To accomplish this objective, electrical potential trends of the ME films under varying low-frequency magnetic fields are investigated and used to demonstrate neural stimulation by calcium imaging on primary neurons in vitro via a capacitive-like charge injection mechanism. In addition, electrical polarization orientation is revealed as a critical design parameter in direct neuron-ME interfaces. These findings collectively demonstrate the potential of nonresonant powering of ME films as a promising minimally invasive wireless neural stimulation technique.

## Teaser

Wireless miniature magnetoelectric films can stimulate neurons, enabling minimally invasive possibilities for neural implants.

## INTRODUCTION

The management of neurological disorders poses a major challenge in an ever-aging society with an increasing number of older adults facing chronic neurological conditions (*1*). Neural implants serve as an alternative set of therapeutic tools, akin to pharmacological agents, for the treatment or management of the neurological disorders. Surgically implanted neurostimulation devices are used under numerous conditions, including movement disorders, chronic pain, and epilepsy (*2*). However, hardware-related challenges, such as dependence on batteries and the risk of infection due to wired designs, remain as a major pitfall for neural implants (*3*). Furthermore, the cost of such devices limits their widespread adoption, especially in the developing countries (*4*). To address these challenges, extensive research has been conducted to develop wireless, minimally invasive, and low-cost interfaces.

In recent years, numerous research has developed wireless neural interfaces of different sizes and demonstrated neuromodulation using different power delivery modalities (*5*–*11*). Light and ultrasound are promising methods for powering neural interfaces; however, they both suffer from absorption or reflection by the tissue and skull and have limited tissue penetration capability compared to magnetic fields (*12*–*16*). The use of alternating magnetic fields (AMFs) for wireless power transfer to target hard-to-reach brain sites, such as deep brain, has a history of more than 60 years (*17*). Although neural implants using radio-frequency electromagnetic waves and near-field inductive coupling have been well studied, tissue absorption and heating remain as safety concerns for implants relying on high-frequency electromagnetic fields (>100 kHz) (*18*, *19*). There is growing evidence for nonelectrical modulation of neural behavior by optogenetic, magnetothermal, and magnetomechanical stimulation methods; nonetheless, reliable translation of these methods into the clinic is challenging due to their dependence on genetic modifications (*10*, *20*, *21*).

To address the safety and efficacy issues, a magnetoelectric (ME) material–based stimulation approach has recently emerged as a promising method (*22*). This approach offers a balanced trade-off between safety and performance because it requires lower carrier frequencies, which results in reduced tissue absorption (*14*). ME materials are typically fabricated in the form of a laminate composite, which consists of magnetostrictive (MS) and piezoelectric (PE) elements (*23*). The ME effect is initiated by the application of AMF and static magnetic field (SMF), which induce strain in the MS element. The mechanical coupling between the MS and PE elements would cause strain to produce stress, which generates charge accumulation on the PE surface (*24*).

The use of ME interfaces at the nanometer-scale and millimeter-scale have been shown as a promising approach for neural stimulation (*8*, *9*, *25*). The use of ME nanoparticles could modulate deep brain circuits in vivo (*8*, *25*). However, reliable use of nanoparticle-based systems is challenging because the effect is dose dependent and colloidal stability, tissue clearance, and accumulation in off-target tissues are potential drawbacks to limit their use in clinics (*26*, *27*). Singer *et al.* explored the use of ME films for deep brain stimulation by designing a device mounted on the skull and wired into the deep brain area (*9*). The design in this work used mechanical resonance frequency as the carrier frequency (>100 kHz) in conjunction with other millimeter-scale ME device designs (*28*–*30*). Despite the ability to generate electric fields exceeding the minimum threshold for neurostimulation, stimulation at a matching frequency is not effective. The resonance frequency is substantially faster than the time constant for the cell membrane; therefore, the membrane potential cannot follow applied frequencies (*9*, *22*, *30*). As a result, such use requires incorporation of additional electronic components to regulate the waveform and frequency, which, in return, increases the volume and complexity of the device. In addition, the dependence on mechanical resonance limits the translational potential of such devices for deep brain applications. It requires even higher frequencies in further miniaturized designs, which limits the safe delivery of energy to the tissue; thus, limiting the tissue depth in which the implant is located. To overcome such limitation, encapsulated designs located at shallow depths and delivering stimulation through wires to the target area have been developed (*9*, *28*, *29*). However, dependence to the wires in such designs does not fully address the problems associated with conventional neural implants. Therefore, development of stable, tunable, and freestanding neural interfaces that operate under nonresonant frequencies to ensure safe operation in variety of biological application scenarios remains as a major challenge for ME neural interfaces.

**Fig. 1.**
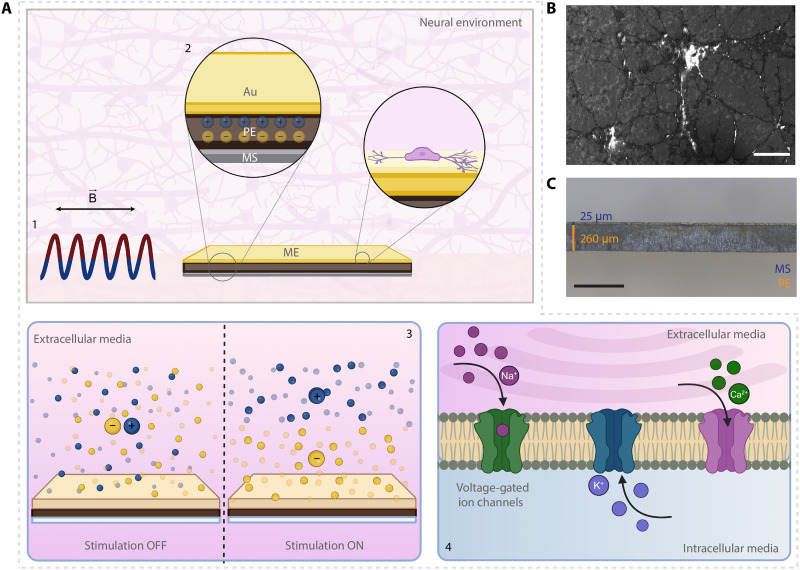
Proposed mechanism of ME neuromodulation using wirelessly powered ME films, consisting of an MS and PE layer. (**A**) Conceptual illustration of the proposed ME neuromodulation mechanism. The gold-coated ME film is powered by an AMF with a superimposed static field (1), which induces charge separation in the PE layer (2). As a result, capacitive-like charge injection occurs in the extracellular environment (3), which causes neuromodulation through the involvement of voltage-gated ion channels (4). (**B**) Scanning electron microscopy (SEM) image of a primary hippocampal neuron grown on an ME film surface. Scale bar, 20 μm. (**C**) Representative side view image of ME films. Scale bar, 500 μm. Created in BioRender. Aydin, A. (2025) https://BioRender.com/roqesvj.

To address the aforementioned challenge of developing safe and controllable ME neural interfaces, here we report the use of millimeter-scale ME films as a direct neural interface for wireless neurostimulation excited in the nonresonant regime (Fig. 1, A and B). Unlike the previous demonstrations, we characterize ME films at frequencies far from resonance and systematically analyze output trends across various AMF and SMF combinations, allowing the identification of optimal signals for neural stimulation. Enabled by our understanding of the system, we present evidence for wireless stimulation of primary hippocampal neurons that can be fine-tuned even at low-frequency weak magnetic fields (10 Hz and 7 mT). Moreover, we provide experimental evidence of electrical stimulation of neurons through calcium imaging experiments in vitro, driven by capacitive-like processes as the primary charge injection mechanism in a biologically relevant environment. Last, we introduce the polarization direction of the PE material as an important design parameter for the use of ME films as a direct neural interface and show its significant impact on the stimulation of primary hippocampal neurons.

## RESULTS

### ME film generates electrical potential wirelessly

ME films were fabricated by combining an MS and PE layer via an adapted protocol published earlier (Fig. 1C) (see Materials and Methods) (*24*). For the electrical characterization, both sides of the PE layer of ME films were attached to electrodes and placed in a one-dimensional (1D) uniform magnetic field generated using custom-built electromagnetic coils (see Materials and Methods). The potential difference between each side of the PE layer was measured by a lock-in amplifier, whereas the amplitude of the SMF was altered (fig. S1) (*31*). The applied magnetic field can be defined as $H_{\text{total}}=h_{0}\left(t\right)+H$ , where H is the amplitude of the SMF and $h_{0}$ is the amplitude of the AMF. Applying an SMF (also referred as bias field) is known to improve generated electrical potentials in ME structures because it offsets the AMF so that the total field oscillates in the region where the strain change in the MS layer is maximized (*9*, *32*).

To optimize geometry of the ME films, the measurements with lock-in amplifier were taken as the basis. First, the samples with fixed long axis length and varying aspect ratios were fabricated (1:1, 1:3, 1:5, and 1:7). In all experiments, long axis of the ME films was aligned parallel to the magnetic field axis. ME film performance was evaluated through determining the ME coefficient ( $\alpha_{\text{ME}}$ ) under varying H while $h_{0}$ was kept as low as 0.01 mT at 10 Hz because a lock-in technique assumes relatively high H values ( $h_{0}$/H ≪ 1) (*31*).

**Fig. 2.**
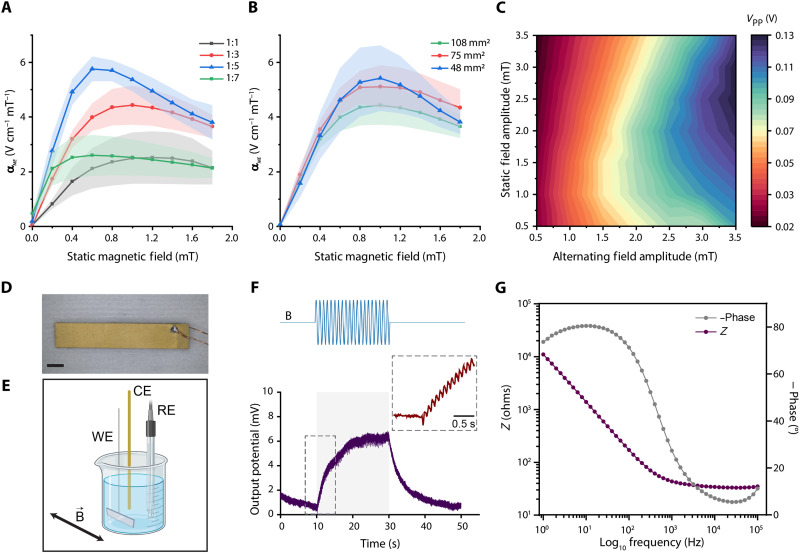
Electrical characterization of the ME films. (**A**) Effect of the aspect ratio (ratio of film width and length) on ME coefficient (α_*ME*_) under varying SMF amplitudes (*n* = 6 from two independent experiments). (**B**) Effect of the ME film area on α_*ME*_ under varying SMF amplitudes (*n* = 6 from two independent experiments). (**C**) *V*_PP_ recorded via an oscilloscope under varying amplitudes of an AMF with a superimposed SMF. (**D**) Representative ME film sample used for electrical characterization. Scale bar, 2 mm. (**E**) ME films are characterized in an electrochemical cell with a three-electrode configuration. The exposed surface of ME films is connected as the working electrode (WE). (**F**) Voltage transient measurement of ME films under 1.6-mT AMF and 1.6-mT SMF at 10 Hz. The shaded area represents the period with the stimulation on. The charge buildup effect is observed as a superimposed 10-Hz sinusoidal signal during the capacitive charging process (inset). Representative sketch of the applied magnetic field (top). (**G**) Electrochemical impedance spectroscopy reading. The frequency was swept between a 1-Hz and 100-kHz sinusoidal input while doing the impedance measurement. The shaded area in the line plots (A and B) represents the SD. Created in BioRender. Aydin, A. (2025) https://BioRender.com/p11y789.

Our results show that a 1:5 aspect ratio generated the best outcome compared to other designs (Fig. 2A). The best operational bias field was found in between 0.5 and 1.5 mT, which varies with the device geometry. Next, we fixed the aspect ratio as 1:3 and evaluated the effect of ME film area on ME performance. Quantitative assessment revealed that the electrode area has no or minimal effect on the ME coefficient in the given range (Fig. 2B).

Next, we assessed maximizing the electrical performance of the ME films under various AMF and SMF combinations. Our results show that increasing both AMF and SMF amplitudes improve the peak-to-peak potential (*V*_PP_) generated by ME films, whereas bias point is shifting at increasing AMF amplitudes (Fig. 2C). However, nonlinear effects dominate under the $h_{0}$ > H condition and creates distortions in the generated potential waveform (fig. S2). Therefore, considering not only the AMF and SMF amplitudes but also their ratio is found to be an important factor for designing the magnetic field profile.

In addition to the standard way of exciting ME films through a sinusoidal magnetic field, we also investigated the performance of the ME films under pulsed magnetic fields (PMFs). For the same peak magnetic field strength, ME films generated a higher *V*_PP_ under rectangular waveform (fig. S3). This could be attributed to the nature of the rectangular wave with quick rise and fall times as the rate change of a magnetic field induces higher strain to the MS layer, yielding stronger electrical response on the PE layer. A maximum *V*_PP_ was observed around the $h_{0}$ = H condition, unlike the sinusoidal excitation case (fig. S4).

We chose the $h_{0}$ = H condition as the condition for the remaining experiments to ensure that the comparisons between conditions were isolated to the amplitudes of the generated signals from the ME films and were not affected by unintended alterations in waveforms or frequencies.

We also observed that the low *V*_PP_ amplitudes from ME films when no SMF is applied can be compensated by increasing AMF amplitudes (fig. S5). However, it should be noted that the frequency of output signal from ME films would be doubled as also observed in the literature (fig. S2) (*33*).

On the basis of the electrical characterization results, we fixed the geometry of the ME film to 3.6 mm by 18 mm by 0.25 mm for the in vitro neural stimulation experiments (Fig. 2D).

### ME films elicit capacitive processes in biological buffers

All biological processes take place in ionic liquid media. Therefore, biological applications require the materials to function in complex and harsh environments whereas the characterization studies are typically conducted under simpler, more controlled dry conditions. Therefore, characterizing biological implants under realistic conditions carries utmost importance to reveal the mechanism of action. Electrochemical cells can provide a similar environment to the neural tissue and allow the characterization of neural interfaces (*34*, *35*). In such characterizations, immediate surrounding of the electrode implanted in the neural tissue can be modeled with the ME films immersed in phosphate-buffered saline (PBS) (*34*) (Fig. 2E). To assess device performance in ionic media, we performed chronopotentiometry (Fig. 2F) and electrochemical impedance spectroscopy measurements (Fig. 2G). The surface impedance of ME films was measured as ~80.74 and ~2580 ohms in biologically relevant frequencies of 200 and 5 Hz, respectively (Fig. 2G). In the same frequency range, the phase angle varies from ~60° up to 80°, which indicates a prominent capacitive nature (Fig. 2G). Validating our observation, voltage transient measurements showed that a charge-discharge curve of the ionic medium–ME film interface displays monophasic and capacitive-like charge injection behavior (Fig. 2F). Moreover, voltage transient measurements revealed the contribution of individual cycles of sinusoidal waves to the accumulated charge and the potential buildup on the neural interface (Fig. 2F, inset).

### Remote powering of the ME films enables neurostimulation in vitro

**Fig. 3.**
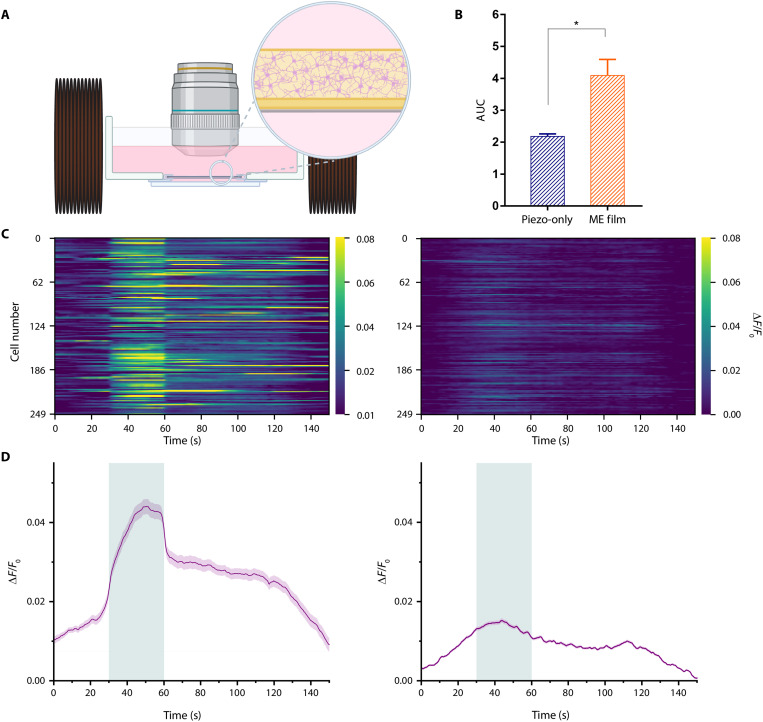
Stimulation of ME films to modulate neuronal activity in vitro. (**A**) In vitro experimental setup for ME stimulation. (**B**) Comparison of the normalized AUC of calcium response during magnetic stimulation under ME and piezo-only conditions (*n* = 3 independent experiments from a total of nine trials under each condition, unpaired two-tailed *t* test, *P* = 0.0179). (**C**) Raster plot of the neural activity from 250 randomly chosen cells in ME film (left) and piezo-only conditions (right). (**D**) Average Δ*F*/*F*_0_ traces from all trials for ME film (left) and piezo-only conditions (right). The shaded area represents the SEM. The bar graph is represented as mean + SEM. The green area represents the stimulation window. Created in BioRender. Aydin, A. (2025) https://BioRender.com/7gvqu4b.

The potential of ME films for neuromodulation was shown through a series of calcium imaging experiments on primary hippocampal neurons in vitro. The neurons were grown directly on the exposed PE surface (Fig. 3A). Before neurostimulation experiments, toxicity was screened through Calcein-AM viability tests (see Materials and Methods). The results showed that the presence of ME films did not induce a prominent toxic effect, with mean viability remaining >95% on the neural cell line at 24-, 48-, and 72-hour-long exposures (fig. S6).

The cells grown on the surface of the ME films were placed in the center of the electromagnetic coils and applied with magnetic fields (fig. S7). To define the stimulation duration, we applied 5-, 15-, and 30-s-long continuous stimulation at the 3.5-mT SMF and 3.5-mT AMF amplitudes. These amplitude combinations were selected as they yielded the highest output potentials without distortion of the waveform. We chose 30 s as the duration of the stimulation for all the experiments because we saw increasing number of cells activated as we increase the stimulation duration from 5 to 30 s (fig. S8).

We investigated the ME films for generating enough electrical potential to modulate neuronal behavior. To test this, we chose the “piezo-only” condition as a control group to observe the baseline activity of neurons because PE films alone should not alter their behavior under a magnetic field. This condition also offers the advantage of maintaining the same neuron-material interface, which helps to ensure that observed effects are attributable to the ME stimulation, eliminating any potential confounding effect.

The exposure to 30-s-long, 10-Hz, 3.5-mT AMF and 3.5-mT SMF induced increased fluorescence intensity of neurons on the ME film unlike the piezo-only case (Fig. 3, B to D, and movie S1). To evaluate the induced bioelectrical effects, we chose the area under curve (AUC) as an outcome measure because it approximates total amount of Ca^2+^ signaling, which is an indicator of the neuronal activity. We normalized AUC during stimulation with before-stimulation reading of the respective experiment to quantify the activity change with stimulation. We found that the increase in the neuronal activity is higher under the ME film condition than the piezo-only condition during magnetic stimulation (Fig. 3B).

### Fine-tuned neurostimulation is achieved with varying magnetic field amplitudes

**Fig. 4.**
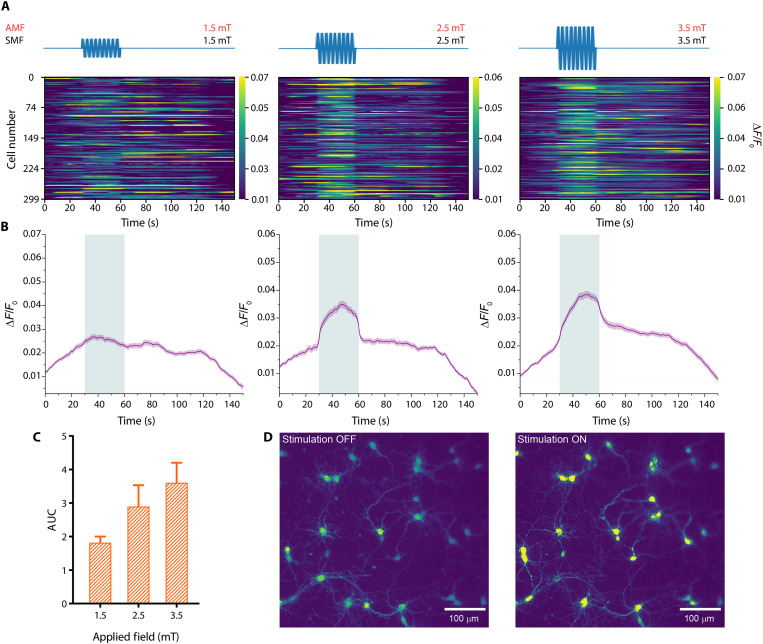
Tuning of neuronal activity with changing magnetic fields. (**A**) Raster plot of the neural activity from 300 randomly chosen cells when 1.5-mT AMF–1.5-mT SMF (left), 2.5-mT AMF–2.5-mT SMF (middle), and 3.5-mT AMF–3.5-mT SMF (right) are applied for 30 s at 10 Hz on the ME film. Representative sketch of the applied magnetic field (top). (**B**) Average Δ*F*/*F*_0_ traces from all trials together for the corresponding 1.5-, 2.5-, and 3.5-mT conditions. (**C**) Comparison of the AUC for increasing magnetic field amplitudes (*n* = 4 independent experiments, from a total of 16 trials under each condition). (**D**) Images depict neurons with average fluorescence levels in the periods before and during stimulation under the 3.5-mT stimulation condition. The shaded area represents the SEM in the line plot. The bar graph is represented as mean + SEM.

Controlling the stimulation intensity is an important aspect of neural stimulation because many applications require tuning of the stimulation. For example, optimization of therapeutic effects and minimizing side effects are balanced through adjusting the stimulation intensity in deep brain stimulation patients (*36*). To account for that, we further tested the effect of applied magnetic field amplitude on the neuromodulation. The magnetic field amplitudes were chosen as 1.5-mT AMF–1.5-mT SMF, 2.5-mT AMF–2.5-mT SMF, and 3.5-mT AMF–3.5-mT SMF to cover the range on the basis of the electrical characterization results (Fig. 2C). Our results showed that the 1.5-mT AMF–1.5-mT SMF condition did not create a robust global increase in neuronal activity (Fig. 4, A and B). However, increasing the magnetic field amplitudes further resulted in a stronger increase in mean fluorescence intensity (Fig. 4C). Under the 3.5-mT AMF–3.5-mT SMF condition, we observed the strongest fluorescence intensities, which indicates global prominent neural activity (Fig. 4, A to D). In the design of this experiment, we paid particular attention to test the same cells under different magnetic field strengths in a given trial and randomized the order of stimulation strengths. Therefore, we eliminated the systematic involvement of short-term plasticity effect, which can cause poststimulation facilitation (*37*). Overall, we can conclude that the application of stronger magnetic fields to the ME film results in stronger stimulation of neurons.

### Direction of PE polarization elicits differential effects on neurons

**Fig. 5.**
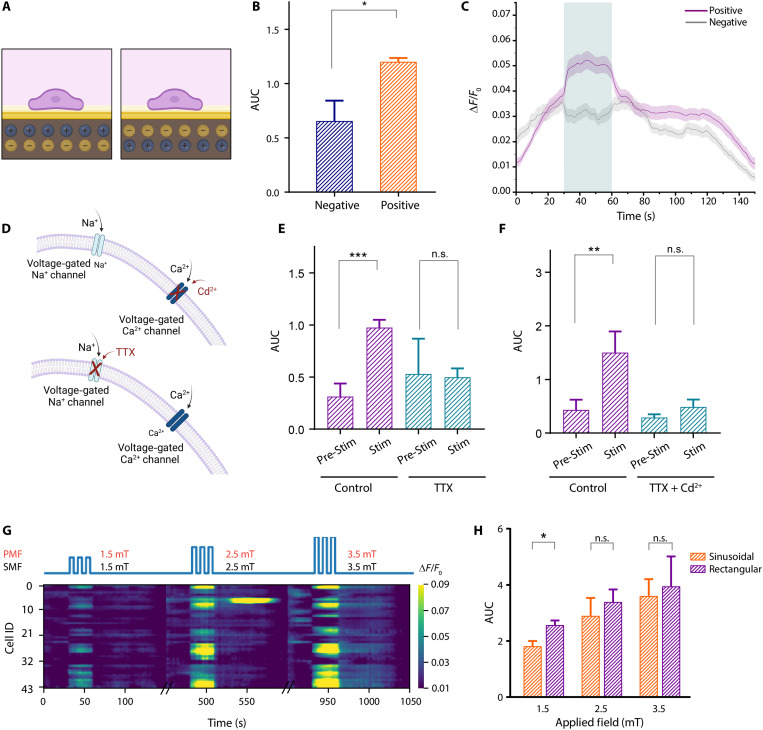
ME neural stimulation, mediated by the voltage-gated sodium and calcium channels, and its dependence on material and magnetic field design. (**A**) Schematic demonstration of a PE polarization direction effect. (**B**) Comparison of the AUC for negative and positive top conditions (*n* = 4 independent experiments, from a total of 12 trials under each condition, unpaired two-tailed *t* test, *P* = 0.0288). (**C**) Average Δ*F*/*F*_0_ traces from all trials for positive and negative top conditions (green shade: stimulation time window). (**D**) Schematic depicting the effect of TTX and Cd^2+^, blocking voltage-gated Na and Ca channels, respectively. (**E**) Neural activity changes before (two-tailed paired *t* test, *P* = 0.0005 and after TTX (0.5 μM TTX) treatment (*n* = 6 independent experiments from 17 trials under each condition, two-tailed paired *t* test, *P* = 0.9372) and (**F**) before (two-tailed paired *t* test, *P* = 0.0037) and after TTX and cadmium treatment (0.5 μM TTX + 200 μM Cd^2+^) (*n* = 6 independent experiments from 16 trials under each condition, two-tailed paired *t* test, *P* = 0.1652). (**G**) Raster plot of a representative trial when 1.5-mT PMF–1.5-mT SMF (left), 2.5-mT PMF–2.5-mT SMF (middle), and 3.5-mT PMF–3.5-mT SMF (right) are applied for 30 s at 10 Hz on the ME film. Representative sketch of the applied magnetic field (top). (**H**) Comparison of the AUC for increasing magnetic field amplitudes comparing sinusoidal and rectangular pulse waveforms [*n* = 4 independent experiments, from a total of 16 trials under each sinusoidal wave condition, of 18 trials from rectangular pulse condition, two-tailed unpaired *t* test, *P* = 0.0239 (1.5 mT), *P* = 0.5499 (2.5 mT), and *P* = 0.7881 (3.5 mT)]. The shaded area represents the SEM. The bar graph is represented as mean + SEM. Created in BioRender. Aydin, A. (2025) https://BioRender.com/br8v456.

Poling is a crucial procedure to use PE materials at their maximum yield. The process aligns electric dipoles of the PE layer, which is oriented axially in our material system. Upon application of a magnetic field, stress is induced on the PE layer, which results in charge separation. We sought to understand the effect of orientation direction in designing ME neural interfaces. For this purpose, we created two groups for each orientation. The positive-top group indicates that cell attachment takes place on the side of the PE layer that accumulates positive charges, whereas in the negative-top group, it is on the side accumulating negative charges (Fig. 5A). Under the same stimulation conditions (3.5-mT AMF–3.5-mT SMF, 30-s duration), an increase in neuronal activity on positive-top was significantly higher than on negative-top (Fig. 5, B and C).

### Neurostimulation is achieved by altering cell membrane potentials

To test our hypothesis that the main mechanism involved for neuronal stimulation is electrical, we conducted a series of experiments under the effect of well-known neurotoxins, namely, tetrodotoxin (TTX), and cadmium (Cd^2+^) to block sodium and calcium channels, respectively. Electrical activation of neurons is driven by a cascade of events that has the direct involvement of voltage-gated sodium and voltage-gated calcium channels (Fig. 5D). A reduced stimulation effect in the presence of these toxins would indicate that the mechanism of activation involves the electrical activation of the cells. To test this, we first measured the activity of cells under 3.5-mT AMF–3.5-mT SMF fields and repeated the same measurement after adding the neurotoxins.

Because addition of neurotoxins altered the baseline condition of neurons, we performed only prestimulation and during-stimulation comparisons. Our results show that, in the absence of any neural blocker, there is a significantly increased neuronal activity during stimulation period, whereas it is diminished upon addition of TTX (Fig. 5E). Similarly, addition of TTX and Cd^2+^ together resulted in reduced stimulation-dependent activation, which is expected to simultaneously block all voltage-gated stimulation pathways (Fig. 5F). Overall, these results indicate that the effect from the ME films involve voltage-sensitive ion channels; hence, it induces electrical modulation of neural behavior.

### PMFs induce stronger neural activation at weaker magnetic fields

We sought to explore versatility of the ME films through providing PMF with 50% duty cycle. The pulse amplitude was chosen as 1.5-mT PMF–1.5-mT SMF, 2.5-mT PMF–2.5-mT SMF, and 3.5-mT PMF–3.5-mT SMF. Similar to the sinusoidal waveform case, the neural activation strength followed the increase in the magnetic field amplitude (Fig. 5, G and H). Moreover, our results revealed that stimulation was consistently more effective in rectangular pulse case with the difference being more prominent at lower field values in line with our characterization findings, which demonstrated a higher *V*_PP_ for rectangular wave pulses compared to sinusoidal waves at the respective field amplitudes (Figs. 2C and 5H and fig. S4).

## DISCUSSION

In this work, we introduced millimeter-scale ME films as a direct neural interface that can provide wireless neural stimulation at adjustable strengths, which is operated at a nonresonant frequency. In addition, we revealed important design considerations for both applied magnetic fields and ME materials, which are crucial for effective use of such devices. As a result, we addressed a set of challenges that had been seen in previous magnetoelectrically powered neural interfaces. These included the dependence on the high-frequency electromagnetic fields (>100 kHz) for operating the millimeter-scale neural stimulation device designs which sets a constraint in the translational potential for the safe use in deep brain sites. Although ME nanoparticle designs address the challenge of high-frequency electromagnetic field requirement, they are limited in the stability along with control of position and electrical field orientation.

Theoretically, it has been shown that increasing the aspect ratio would increase the electrical potential output due to the magnetic flux concentration effect (*38*). We have seen this trend experimentally until 1:7, which might be attributed to the limitations of our fabrication method, such as the impact of heat on PE polarity (see Materials and Methods). In addition, it is theoretically shown that the ME coefficient ( $\alpha_{\text{ME}}$ ) is independent of area, which we verified experimentally (*39*). Our results also ensure that we operate far enough from resonance because the length change in ME film did not induce a considerable effect in $\alpha_{\text{ME}}$.

The presence of dominant capacitive behavior in ME films is particularly important because a capacitive charge injection mechanism is favored for neural stimulation purposes because it provides safe and efficient charge injection (*35*). Meanwhile, nonreversible faradaic interfacing with the medium has a potential to cause a reaction on the electrode surface and proteins (*15*, *40*). We argue that the potential buildup through the contribution of individual cycles of sinusoidal waves on the neural interface can be also supported by findings from electrochemical characterization of ME nanodiscs, where a similar monophasic charging curve is observed (*25*). Similarly, we observed that the amount of charging and discharging is not symmetric in a sinusoidal cycle in our previous study with ultrasound-powered PE materials interacting with sinusoidal excitation and modulating neural activity (*11*).

In our study, we found that PE polarization direction is an important design parameter. We believe that the effect can be explained by the complex interplay of ME effect and neuronal behavior. In our material system, MS layer (Metglas) would be elongating under an external magnetic field because it has a positive magnetostriction coefficient. This could be causing stress-relaxation cycles in each sinusoidal cycle on the PE layer. However, the direction of the forces would not reverse during the negative half of the sinusoidal cycle; instead, the applied stress diminishes without inverting the direction, causing asymmetric surface potentials and charge accumulation over time in the liquid–ME film interface (in the case of $h_{0}$ < H ). In the context of extracellular neural stimulation, electric fields generated by the PE layer would be causing ions in the liquid environment to rearrange to screen the generated electric field; therefore, ions would be rearranged within each sinusoidal cycle. Considering the two-domain stimulation model suggested by Schoen and Fromherz, the direction of capacitive current (positive-top versus negative-top) would affect local ion distribution and membrane polarization differently (*41*). In their experimental design, where the neuron adhesion on an insulated planar electrode takes place, rising and falling voltage ramps are delivered (corresponding to positive and negative charge accumulation in our system, respectively). The dependence on polarization direction in our system might stem from their following findings; primary capacitive depolarization of free membrane is induced by rising voltage ramps via hyperpolarization of the attached membrane, and depolarization of the membrane attached to an electrode is induced by the falling ramps. The latter is more likely to fail because the ion current through the attached membrane might not be sufficient to elicit an action potential, due to the relatively small area of the attached membrane compared to the free membrane (*41*). On the other hand, further studies with concentrated characterizations could provide detailed understanding to verify this hypothesis. Regardless of the exact mechanism behind this effect, we believe that this finding presents a crucial consideration in designing ME films as direct neural stimulation interfaces.

Overall, our experiments provide multiple evidence for electrical stimulation of neurons. Although neurotoxin studies demonstrate the involvement of voltage-gated ion channels, the dependence of neuromodulation on the polarization direction also provides another proof eliminating nonelectrical neuromodulation mechanisms, such as mechanical and thermal stimulation, as the mechanical and magnetic properties of the MS layer would be identical in both configurations. In addition, the saturation magnetostriction of Metglas is only 25 to 40 parts per million, which is orders of magnitude less than the strains required for calcium influx via Piezo1 channels in similar systems where the entire cell body is subjected to uniaxial strain (*42*–*44*).

We observed more effective neural stimulation with ME films excited by PMFs, compared to sinusoidal excitation. We believe that it can be attributed to the fast potential rise component with a larger *V*_PP_. However, such use might be more complex because using magnetic pulses safe and reliably is more challenging than sinusoidal waves for reasons, such as pronounced generation of harmonics. Therefore, the mechanism and transferability of such use of ME films could be enlightened through further studies.

Translation of such ME interfaces for specific clinical scenarios still faces multitude of challenges. Our current in vitro system operates while neurons are located at a very short distance from the electrode surface, with similar systems reporting the range of tens of nanometers (*41*). At tissue-level applications, miniaturization and alternative design methods, such as electrode surface functionalization and integration of biomimetic approach that aims matching mechanical properties of the neural tissue, might be helpful to use the ME approach further (*45*–*47*). Furthermore, long-term stability and toxicity are major concerns, as with other implants. Although we did not see a prominent toxic effect in our experimental work, lead-free materials with comparable PE coefficient would be ideal to ensure long-term safety of such devices (*48*). In addition, to ensure material stability in harsh environments, we coated the MS side of ME films with parylene C, which is a common polymeric coating that is found in current biomedical devices designs. Long-term toxicity studies are needed to understand the full capacity of ME films (*49*).

In our study, we have shown that there is charge accumulation over time in each sinusoidal cycle at the neural interface. Because we already use a nonresonant paradigm, the generated *V*_PP_ in ME is less dependent on driving frequency (*8*). However, this gives a room to manipulate the overall injected charge by changing the driving frequency to increase the number of cycles in the given stimulation period and stimulation duration depending on the exact application scenario. This can also help addressing the overall reduction in power in miniaturized designs. Translation of such interfaces would require setting its own stimulation parameters depending on the exact application scenario; therefore, we believe that benchmark electrical potential and current values from conventional electrodes may not be directly applicable.

Translation of ME neural stimulation technologies would be important in the future not only for its potential hardware-related benefits due to its freestanding design but also from a broader perspective of patient accessibility. Now, the cost of invasive neural stimulation therapies is the bottleneck for widespread adoption of such therapies, especially in developing countries (*4*). Limited battery life requires multiple hospital admissions, which not only cause discomfort and potential surgical complications for patients but also substantially increase the overall cost of care. In addition, the cost burden of the hardware is mainly defined by the implantable pulse generator, which could be addressed through wireless designs. For the future applications, our proposed system could potentially provide a low-cost alternative as it requires a rather simple design of electromagnetic coils and implanted device.

In summary, we demonstrated that ME films can be used as direct neural interfaces without needing any additional electronics on board. By designing the geometry of ME films and input magnetic fields, we established that they can generate enough power to modulate activity of neurons wirelessly in nonresonant frequencies, which is proven through in vitro calcium imaging experiments. In addition, we presented evidence to enlighten the stimulation mechanism. We demonstrated that the stimulation is through involvement of sodium and calcium channels, which indicates the presence of electrical neuromodulation. Future work will be conducted to tackle translational challenges of ME films and explore potential long-term use of them in vivo.

## MATERIALS AND METHODS

### Fabrication of ME films

We fabricated the ME films by laser cutting Metglas (2605HB1M, Metglas Inc.) (MS layer) and prepolarized and metallized PE lead-zirconate-titanate (PZT) plates (M1100, Hoerbiger) (PE layer) into desired dimensions. After cutting, the films were laminated to each other with epoxy (M-Bond-600, Micro Measurements) and cured at 125°C for 4 hours in an oven. In the electrical characterization experiments, copper wires were soldered to the PE plates on both sides before the bonding to the Metglas. PZT plates are delivered as polarization orientation indicated by the manufacturer. To fabricate ME films with different PE polarization orientations, we adhered Metglas to either surface of the PZT plates.

For in vitro neuromodulation experiments, ME films were fabricated into 3.6 mm–by–18 mm dimensions. Before the seeding of the neurons in all in vitro experiments, ME films were coated with parylene C (PDS 2010, SCS) via vapor deposition to protect them from a harsh biological environment. During the coating, a mask was applied to the PE surface and removed after the coating to make sure to keep the active surface exposed for the cell attachment.

### ME film characterization

ME films are characterized for the effect of geometrical properties using the setup in fig. S1. The setup comprises two isocentric Helmholtz coils to generate an AMF and SMF and a lock-in amplifier (SR830, Stanford Research Systems) as the voltage measurement system. Coils were shielded for both low-frequency and high-frequency electromagnetic fields with high-μ material (MU Metal Foil, PSE-Priggen) and aluminum to minimize the electrical interference from coils with the measurement system. The first pair of coils is connected to an arbitrary signal generator through a linear voltage amplifier that provides an AMF up to an amplitude of 2 mT, and a dc power supply powers the second pair of coils to generate the static bias magnetic field up to 1.8 mT. The ME films were clamped on one end and placed in a small aluminum casing, which functions as a Faraday cage to mitigate the inductive effect of the coils’ electromagnetic field on the clamp connections. The casing is placed in the center of the Helmholtz coils, and the clamped faces are connected to the differential lock-in amplifier using shielded microminiature coaxial (MMCX) wires and connectors. A calibration routine was performed before each experiment by measuring the resulting magnetic field (460, LakeShore) and current flow (U1273A, Keysight). Results are reported as the ME coefficient ( $\alpha_{\text{ME}}$ ), which is calculated as

$$\alpha_{\text{ME}}=\frac{V_{\text{out}}}{h_{0}d}$$

where $V_{\text{out}}$ is the differential voltage measured by the lock-in amplifier, $h_{0}$ is the AMF amplitude, and d is the PE film thickness (*31*).

### Electrochemical characterization

Electrochemical characterization experiments were performed using the Autolab potentiostat/galvanostat PGSTAT (Metrohm). A two-electrode configuration was used for surface impedance measurements. ME films were connected to the working electrode on the exposed side of the PE layer, whereas the Ag/AgCl electrode was used as a reference electrode. Electrical potential measurements were carried out in a three-electrode configuration, with the addition of platinum wire as the counter electrode into the system. To excite the ME films, the electrochemical cell was placed in the center of the electromagnetic coils. The ME film is positioned in the electrochemical cell as the longest dimension aligned parallel with the applied magnetic field axis.

### Fabrication of the electromagnetic coil system for in vitro experiments

The custom-built Helmholtz coil system was fabricated to generate uniform magnetic fields in the center of two 9.5-cm-diameter coils (fig. S8). The frames of the coil system were designed in SolidWorks and 3D-printed using a Form3 3D printer (Formlabs). The material of the coil frame is High Temp Resin (Formlabs), which has a high heat deflection temperature of 238°C. The 1.5-mm-thick enameled copper wire was wound around coil frame. Each coil was driven by an independent motor driver acting as a current controller (Maxon ESCON 70/10). The power for the current controllers was supplied by Mean Well SDR-960-48 (48-V dc at 20 A). The two motor drivers were connected to the analog output channels of a National Instruments USB-6363, which was controlled by a LabView program on a PC. The dynamic performance of the current controllers was tuned manually in the vendor’s software Maxon Studio. Each coil was independently calibrated by measuring the magnetic field (B) in five locations in the workspace. The measured B was used to calculate the current–to–B matrix (*50*). The coil system was used in all in vitro experiments, peak-to-peak electrical potential, and electrochemical measurements.

### Culture of primary hippocampal cells

Primary rat hippocampal neurons (Thermo Fisher Scientific, Gibco, A36513) were prepared according to the manufacturer’s recommendations. Before plating for experiments, ME films were coated with poly-d-lysine (10 μg/ml; Sigma-Aldrich, P6407) and laminin (5 μg/ml; Sigma-Aldrich, L2020) in PBS (Thermo Fisher Scientific, Gibco, 10010023) for at least 1 hour in the incubator. Coverslips were then washed three times thoroughly with distilled water. Frozen cells from liquid nitrogen were rapidly thawed by gently swirling in a 37°C water bath. The cryovial was washed once with 1 ml of complete medium consisting of Neurobasal Plus Medium (Thermo Fisher Scientific, Gibco, A3582901) supplemented with 0.5 mM GlutaMAX-I, B27 Plus (Thermo Fisher Scientific, Gibco A3582801), and 25 μM l-glutamate (TMS-002-C, Sigma-Aldrich). The neurons were suspended to a total volume of 4 ml of complete medium. Viable cell density was determined with a hemocytometer (DHC-N01, NanoEntek). The cell suspension was diluted accordingly, and cells were plated at a density of 50,000 cells/cm^2^. Cells were incubated at 37°C in a humidified atmosphere of 5% CO_2_ in air. The medium was half refreshed 24 hours after incubation by aspirating half of the medium and replacing with a fresh medium. From day 4 on, the medium was half refreshed every 3 days with the abovementioned medium without 25 μM l-glutamate. The calcium imaging experiments were performed between 7 and 14 days after plating of the cells, where the cell maturation was observed through formation of dendrites and branches.

### ReNcell culture

The human-derived neural progenitor cell line (ReNcell VM, SCC008, Sigma-Aldrich) was cultured in ReNcell NSC Maintenance Medium (SCM005, Merck) containing fibroblast growth factor 2 (20 ng/ml; GF003, Sigma-Aldrich) and epidermal growth factor (20 ng/ml; GF144, Sigma-Aldrich). The culture was maintained in a humidified incubator with temperature control at 37°C and CO_2_ control at 5%. The ReNcell culture was subcultured at the ratio of 1:5 once it reached 90% confluence. Briefly, cells were washed with Dulbecco’s phosphate-buffered saline (DPBS)without calcium or magnesium (Thermo Fisher Scientific, Gibco 14190250) and detached from the flask by using Accutase (A6964, Sigma-Aldrich). Accutase was deactivated by addition of a maintenance medium minimum two times of the volume of the Accutase. Neurons were centrifuged at 300*g* for 4 min. The cell pellet was then resuspended in a culture medium, and the cell concentration was counted using a hemocytometer (DHC-N01, NanoEntek). For biocompatibility tests in vitro, cells were plated in laminin-coated [L2020, Sigma-Aldrich at 20 μg/ml in DMEM (Dulbecco’s modified Eagle’s medium)/F-12 medium, 11320033, Thermo Fisher Scientific] cell culture–treated 96-well plates at a density of 40,000 cells/cm^2^.

### Calcium imaging experiments

ME films with primary neurons on the surface were incubated in 1 μM Fluo-4 AM dye (F14201, Thermo Fisher Scientific) in Hanks’ balanced salt solution (HBSS; Thermo Fisher Scientific) for up to 60 min at room temperature in the dark as suggested in the product protocol. After loading, cells were washed with HBSS, and imaging was conducted in HBSS + 20 μM glucose solution. Fluo-4 was excited using a 460-nm LED (light-emitting diode), and time-lapse images were recorded at ×20 magnification every 0.2 s and recorded using a Prime BSI Scientific CMOS (sCMOS) digital camera.

ImageJ software was used for preprocessing steps such as motion correction, background subtraction, and ROI (region of interest) selection. A Python script was adapted from the literature to carry out moving average implementation and Δ*F*/*F*_0_ calculation on the Ca^+2^ transient data where *F*_0_ is defined on the basis of the minimum local baseline of the moving average window (*10*). Ca^2+^ transients are demonstrated as Δ*F*/*F*_0_. AUC calculations were conducted on Δ*F*/*F*_0_ curves. For AUC calculations, the minimum value of the first 60 s of each trace (until the end of the stimulation phase) was taken as baseline. Except for the neurotoxin experiments, the during-stimulation condition was normalized to the prestimulation AUC of the respective trace to eliminate the batch to batch variation of staining and neural behavior. AUC calculations and statistical tests are conducted on GraphPad Prism software. The cells were defined as active if the four times of SD from Δ*F*/*F*_0_ curves of prestimulus recordings are exceeded during stimulation.

Images in Fig. 2D were generated by subtracting the average of the prestimulus period from the whole stack and creating an average in both prestimulus and during stimulation periods through the Z-project function of ImageJ.

In the channel blocker experiments, first baseline recordings were performed. Subsequently, the chemical blockers were directly introduced to the extracellular medium. To pharmacologically block voltage-gated sodium channels, TTX was applied at 0.5 μM as it is reported to exert a nontoxic but inhibitory effect (*11*). Last, 200 μM cadmium chloride (Cd^2+^) was introduced to neurons to block voltage-gated Ca^2+^ channels (*11*). Addition of blockers was observed to affect prestimulation transients; therefore, comparisons were carried out between prestimulation and during stimulation of baseline and blocker recordings without a normalization.

### Biocompatibility assays

Neurons from ReNcell cell line were seeded to cell culture–treated 96-well plates for biocompatibility tests. A day after seeding the cells, ME films (5 mm by 5 mm) were introduced to wells. The wells with neurons and without ME film were defined as the control group. The cell viability was measured using the LIVE/DEAD cell imaging kit (R37601, Invitrogen, Thermo Fisher Scientific) at 24, 48, and 72 hours after the addition of ME films. Staining was performed according to the manufacturer’s recommendations. The images were captured with a Nikon spinning disc confocal microscope (Nikon Eclipse Ti-E). The cell counting was performed in ImageJ software through thresholding, watershed, and analyze particle functions. The viability results were reported as normalized to the respective control group.

### Statistical analysis

All quantitative values are presented as means ± SEM unless stated otherwise. Two-tailed Student’s *t* tests were performed for two group comparisons by using GraphPad Prism 6 (GraphPad Inc.) (Figs. 3B and 5, B, E, F, and H, and fig. S6B). If the same neurons are assessed in group comparisons, the paired *t* test is used. *P < 0.05 was considered as statistically significant, and nonsignificant differences are presented as “n.s.” In each figure, *P* values were represented as *P < 0.05, **P < 0.01, ***P < 0.001, and ****P < 0.0001.
