# Supplementary material for: Wireless nonresonant stimulation of neurons on a magnetoelectric film surface
Source: Sci Adv. 2025 Oct 17;11(42):eadx6829. doi: 10.1126/sciadv.adx6829 (PMC12533637; doi:10.1126/sciadv.adx6829)
Supplement: Supplementary file 1 — Figs. S1 to S8 Legend for movie S1 [file sciadv.adx6829_sm.pdf]

Science Advances
AAAS

Supplementary Materials for

Wireless nonresonant stimulation of neurons on a magnetoelectric film surface

Asli Aydin *et al.*

Corresponding author: Metin Sitti, msitti@ku.edu.tr

Sci. Adv. **11**, eadx6829 (2025)
DOI: 10.1126/sciadv.adx6829

The PDF file includes:

Figs. S1 to S8
Legend for movie S1

Other Supplementary Material for this manuscript includes the following:

Movie S1

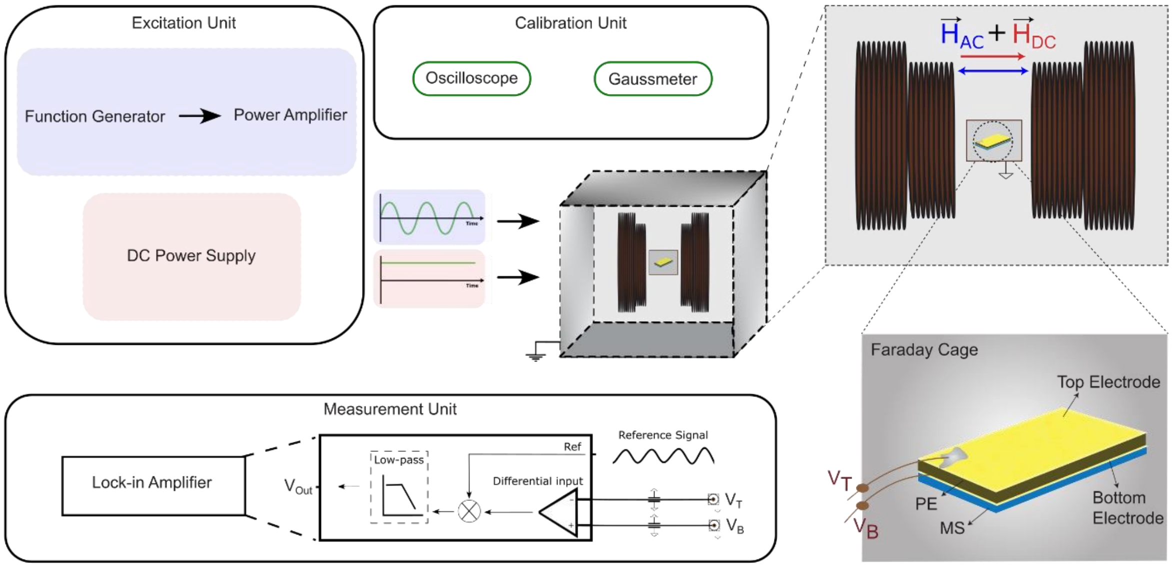

Fig. S1. Experimental schematic of lock-in characterization setup. Schematic of electromagnetic coil setup used for magnetoelectric coefficient-based characterizations. A 2-coil system enables the generation of superimposed alternating and static magnetic fields. The coil system is shielded against low and high frequency magnetic fields to avoid the effect of coils on the measurement system. ME films are shielded against the inductive effect from coils. The measurements were recorded in differential mode by a lock-in amplifier.

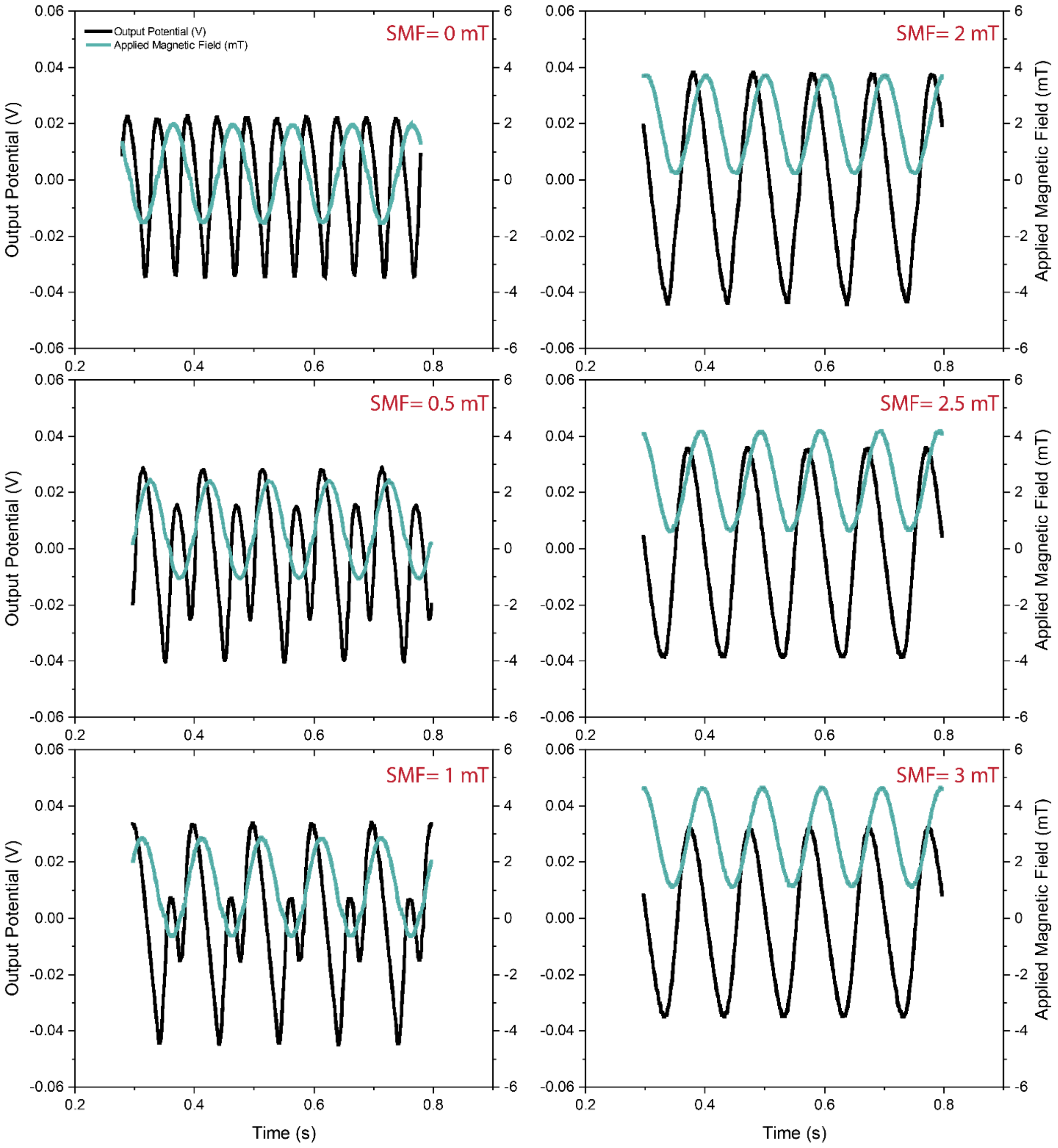

Figure S2. Effect of static magnetic field on ME film output potentials. Exemplary traces from ME film output potential measurement with oscilloscope. 2 mT AMF is superimposed on varying SMF amplitudes. Non-linear ME effect can be seen in under $h_0/H > 1$ field, which vanishes as the static magnetic field amplitude increases.

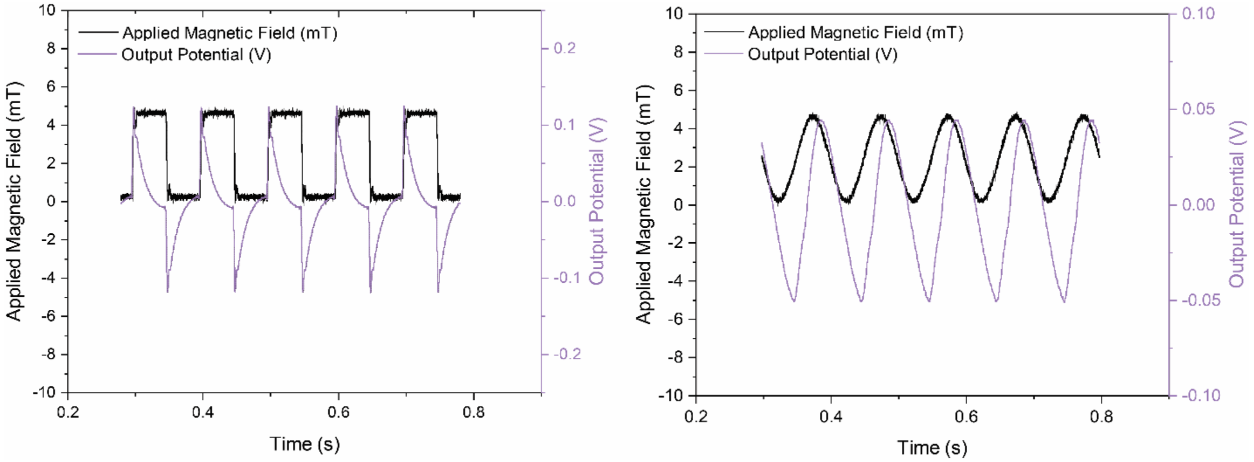

Figure S3. Electrical potential generation from ME films under pulse and sinusoidal magnetic fields. Exemplary trace of ME film output potential by oscilloscope under rectangular pulse and sinusoidal magnetic field. 2.5 mT SMF is superimposed on 2.5 mT PMF (left), and 2.5 mT SMF is superimposed on 2.5 mT AMF with sinusoidal waveform (right). Recorded V_{PP} is found to be higher in the pulsed magnetic field condition. Magnetic field traces are measured by a gaussmeter and read on an oscilloscope.

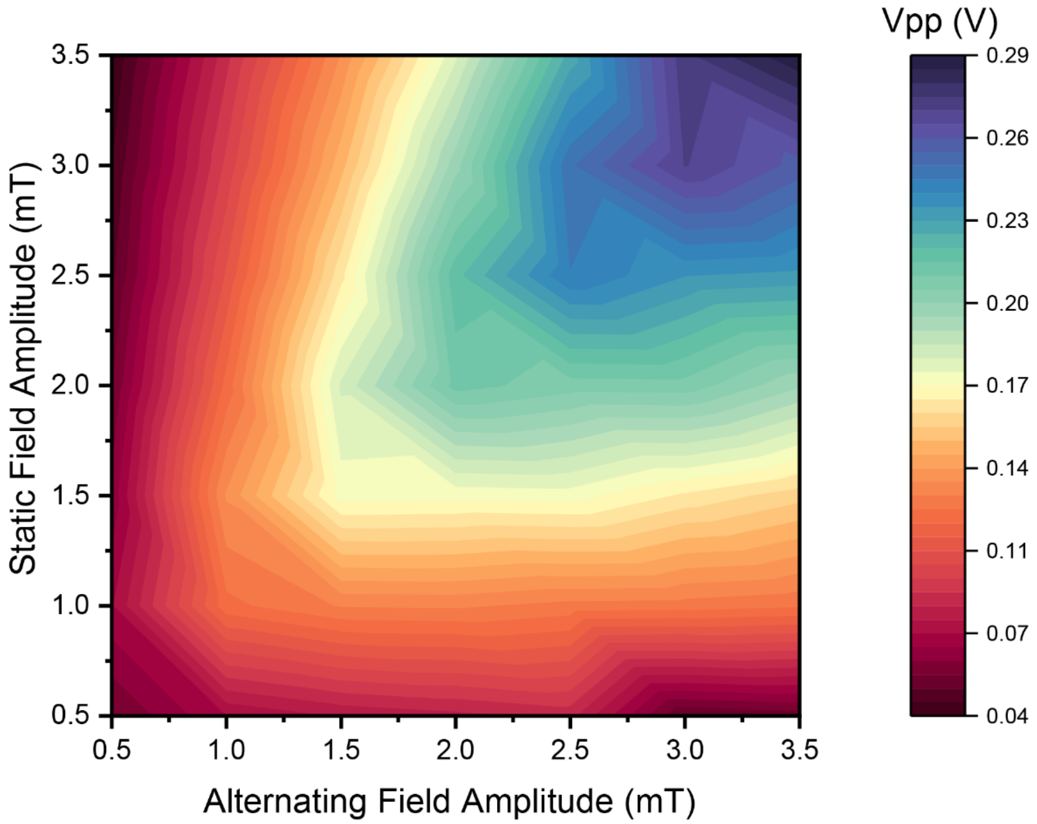

Figure S4. Peak-to-peak potential readings under varying amplitudes of PMF with superimposed SMF. Peak-to-peak potential (V_{PP}) readings reveal higher V_{PP} generation in comparison to the application of sinusoidal AMF. Moreover, the contribution of the static magnetic field on V_{PP} is maximized when its amplitude is closer to the pulse amplitude.

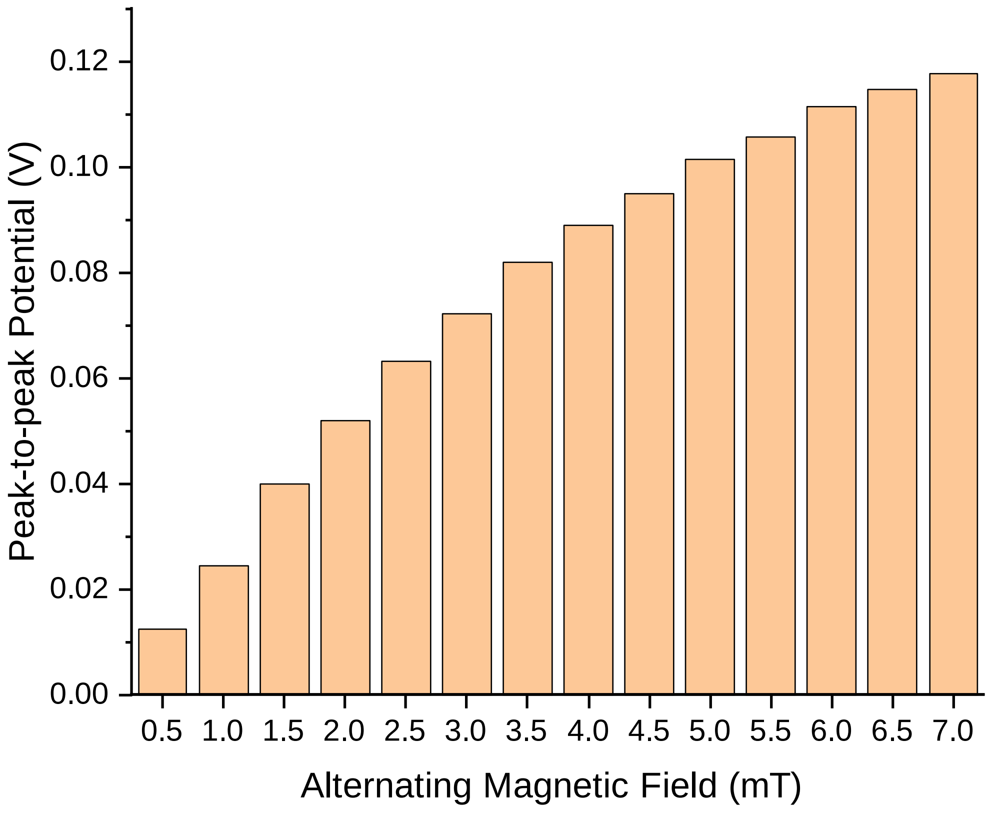

Figure S5. ME film peak-to-peak potentials under increasing alternating magnetic fields, without a static field. Peak-to-peak potential readings reveal higher potential generation as the AMF amplitude increases.

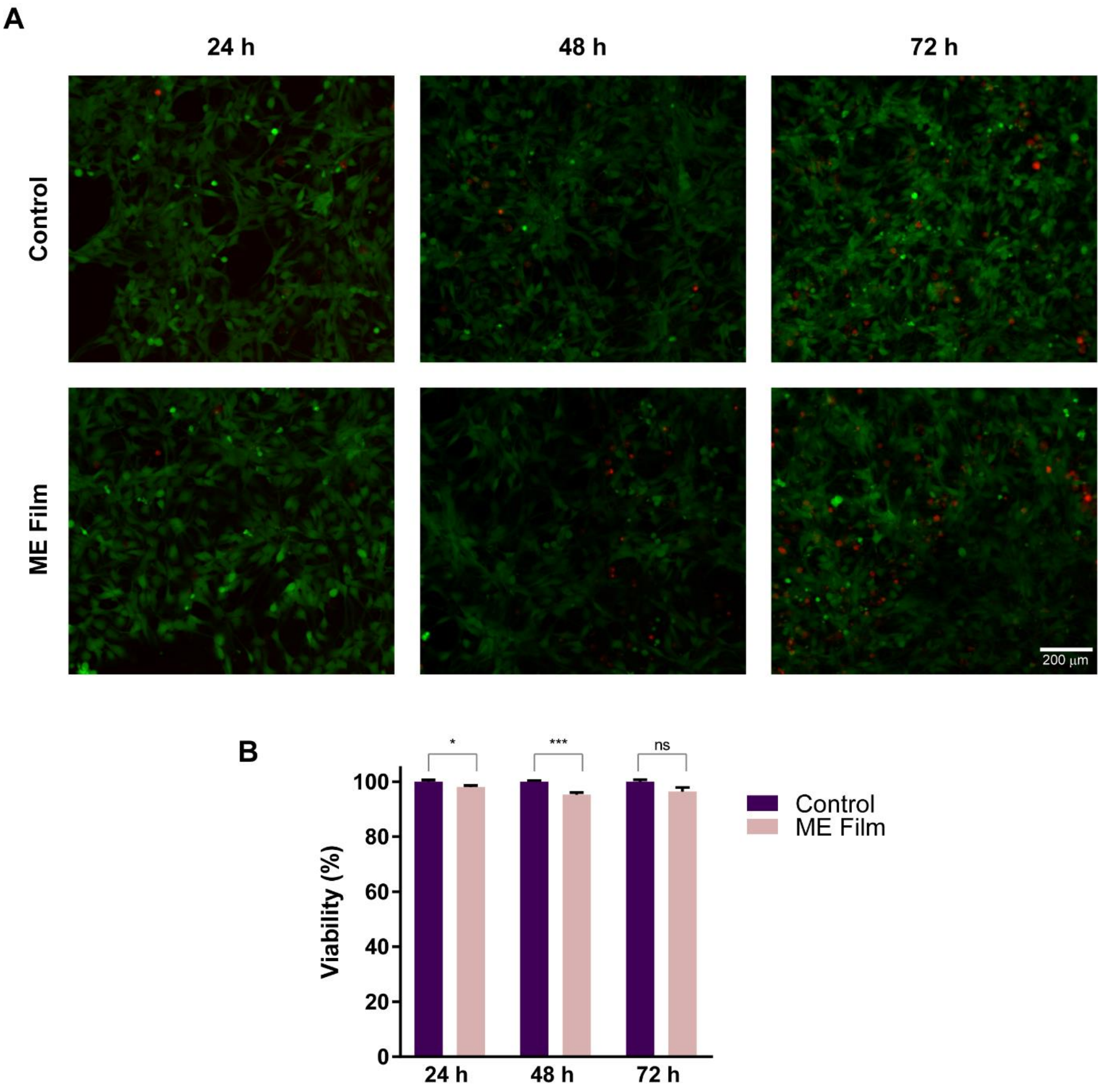

Figure S6. Toxicity screening of ME films. A LIVE/DEAD™ cell imaging of RenCell cells at 24, 48, and 72 hours. The green color indicates live cells, the red color indicates dead cells. **B** Cell viability (%) results are reported as normalized to the control group of the corresponding condition ($n = 6$, from 2 independent experiments, unpaired two-tailed t-test, 24h: $p = 0.0325$, 48h: $p = 0.003$, 72h: $p = 0.0509$). Bar graph is represented as mean + s.e.m. *$p < 0.05$ was considered statistically significant and ns: not significant.

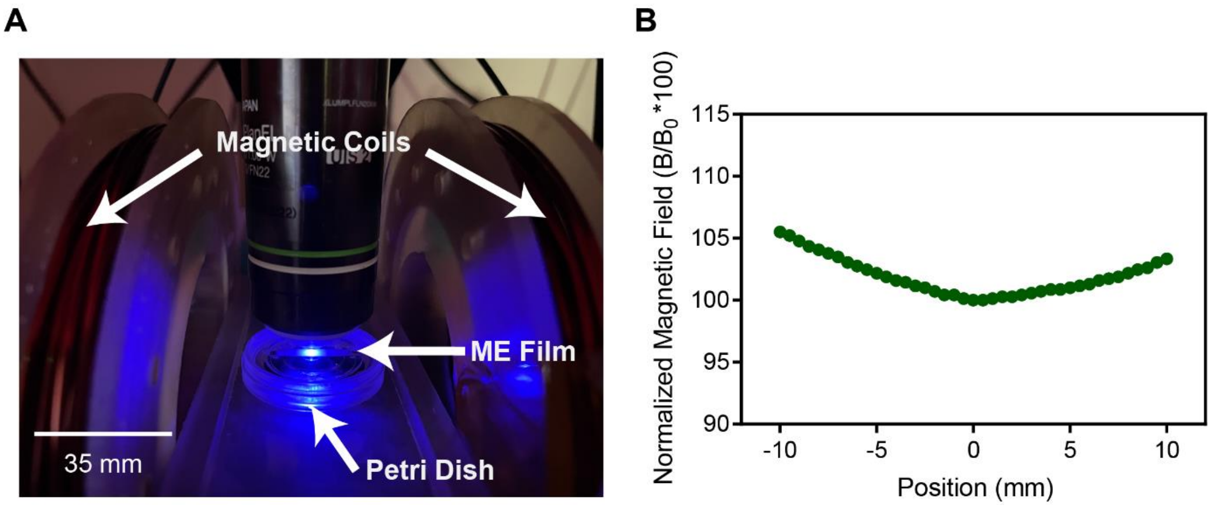

Figure S7. In vitro magnetic stimulation setup. A In vitro magnetic stimulation setup for magnetoelectric neuromodulation. **B** Magnetic field gradient measurement is recorded 7 mm above from the coil center, along the field axis. The measurement is reported as normalized to the field amplitude in the centre of the coils (B_0).

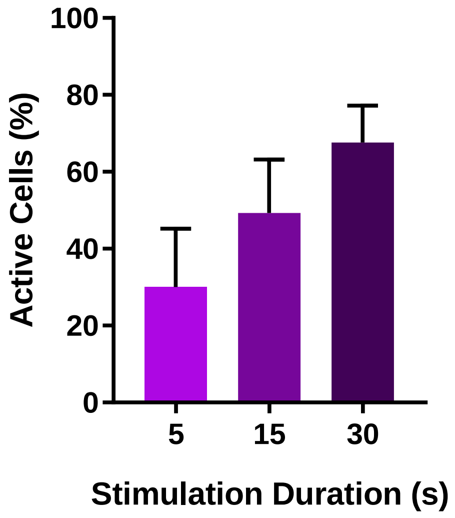

Figure S8. Effect of stimulation duration on neuronal recruitment. Comparison of active cell ratio under increasing pulse durations at 3.5 mT AMF and 3.5 mT SMF, 10 Hz on ME film. (n=3 independent experiments, from a total of 9 trials in each condition). Bar graph is represented as mean + s.e.m.

Description of supplementary movies

Movie S1. In-vitro assessment of magnetoelectric neural stimulation through calcium imaging. This video contains calcium imaging of primary hippocampal neurons grown on ME film surface before, during, and after the application of 3.5 mT SMF and 3.5 mT AMF at 10 Hz. The attached video is further processed in Image J for visualization purposes to account for high baseline intensities and photobleaching effects.
